# Pathophysiological Responses in Rat and Mouse Models of Radiation-Induced Brain Injury

**DOI:** 10.1007/s12035-015-9628-x

**Published:** 2016-01-22

**Authors:** Lianhong Yang, Jianhua Yang, Guoqian Li, Yi Li, Rong Wu, Jinping Cheng, Yamei Tang

**Affiliations:** 1 Department of Neurology, Sun Yat-Sen Memorial Hospital, Sun Yat-sen University, Number 107, Yan Jiang Xi Road, Guangzhou, Guangdong Province 510120 China; 2 Key Laboratory of Malignant Tumor Gene Regulation and Target Therapy of Guangdong Higher Education Institutes, Sun Yat-sen University, Guangzhou, 510120 China; 3 Department of Neurology, Fujian Provincical Quanzhou First Hospital, Quanzhou, Fujian Province China; 4 Guangdong Province Key Laboratory of Brain Function and Disease, Zhongshan School of Medicine, Sun Yat-sen University, 74 Zhongshan 2nd Road, Guangzhou, 510080 China

**Keywords:** Radiation, Brain injury, Pathogenic mechanism, Animal model

## Abstract

The brain is the major dose-limiting organ in patients undergoing radiotherapy for assorted conditions. Radiation-induced brain injury is common and mainly occurs in patients receiving radiotherapy for malignant head and neck tumors, arteriovenous malformations, or lung cancer-derived brain metastases. Nevertheless, the underlying mechanisms of radiation-induced brain injury are largely unknown. Although many treatment strategies are employed for affected individuals, the effects remain suboptimal. Accordingly, animal models are extremely important for elucidating pathogenic radiation-associated mechanisms and for developing more efficacious therapies. So far, models employing various animal species with different radiation dosages and fractions have been introduced to investigate the prevention, mechanisms, early detection, and management of radiation-induced brain injury. However, these models all have limitations, and none are widely accepted. This review summarizes the animal models currently set forth for studies of radiation-induced brain injury, especially rat and mouse, as well as radiation dosages, dose fractionation, and secondary pathophysiological responses.

## Introduction

Radiation-induced brain injury is a continuous and dynamic process. Based on the time course of clinical expression, radiation-induced brain injury can be classified into the following three phases [1]. (1) Acute reactions, which occur within 2 weeks after the beginning of radiotherapy and sometimes during the course of irradiation. Patients may experience nausea and vomiting, headache, fatigue, increased neurological symptoms and signs, and even death due to brain herniation. (2) Early delayed reactions, which develop 2 weeks to 6 months after irradiation. These complications may be related to transient demyelinating processes associated with blood-brain barrier (BBB) disruption or to selective oligodendrocyte dysfunction and include somnolence syndrome, deterioration of pre-existing symptoms, transitory cognitive impairment, and subacute rhombencephalitis/brain stem encephalitis. (3) Late delayed reactions, appearing several months to years after radiotherapy, including focal brain necrosis and mild to moderate cognitive impairment. Late delayed reactions are irreversible and devastating and thus are of major concern.

The mechanisms of radiation-induced brain injury corresponding to clinical manifestations are not fully understood. Two theories have been advanced [2]. The first proposes that the most severe consequences of irradiation result from direct impairment of brain parenchymal cells, while changes to the vasculature are of comparatively minor importance. The second proposes that radiation-provoked damage to the vascular system is of paramount importance and leads to brain ischemia. To explore the underlying mechanisms of radiation-induced brain injury and the suppositions of the two theories, researchers have established animal models to investigate the pathogenesis and histopathology of the injury state [3]. Initially, numerous types of animal species were utilized in these experiments, including dogs and monkeys as early as the 1930s. It was not until the 1960s that large-scale experimentation with rats and mice was initiated [4].

Ideally, primates are the most appropriate candidates to model radiotherapy-provoked human disease, taking into account radiosensitivity and the radiation threshold. However, primate research is expensive, and not without ethical concerns. Given their genetic background, anatomical structure, operability, and relatively low cost of use, rats and mice now provide some of the most advantageous models of human disease and injury states.

At the advent of research into radiation-induced brain injury, animal models were used to uncover assorted pathological changes (e.g., vascular lesions, edema, necrosis, and demyelination). However, animal models were soon applied to behavioral science investigations as well because cognitive dysfunction is now recognized as one of the most common late effects of radiotherapy [5]. More recently, the availability of molecular and genetic tools and new insights in neurobiology propelled the discovery of more nuanced responses to radiotherapy at the cellular and molecular levels. At the same time, subtle cellular and tissue changes were observed with relatively low doses of radiation, in addition to radiosensitivity in different regions of the brain.

This review addresses the influence of radiation dose, fractionation, volume, and other parameters on functional and histopathological changes in animal models of radiation-induced brain injury, with a focus on rats and mice. To our knowledge, this is the first review on the subject matter. We anticipate that our summary of the literature to date will provide critical information driving further animal studies on radiation-provoked pathologies.

## Animal Models of Radiation-Induced Brain Injury

### Rats

**Fig. 1:**
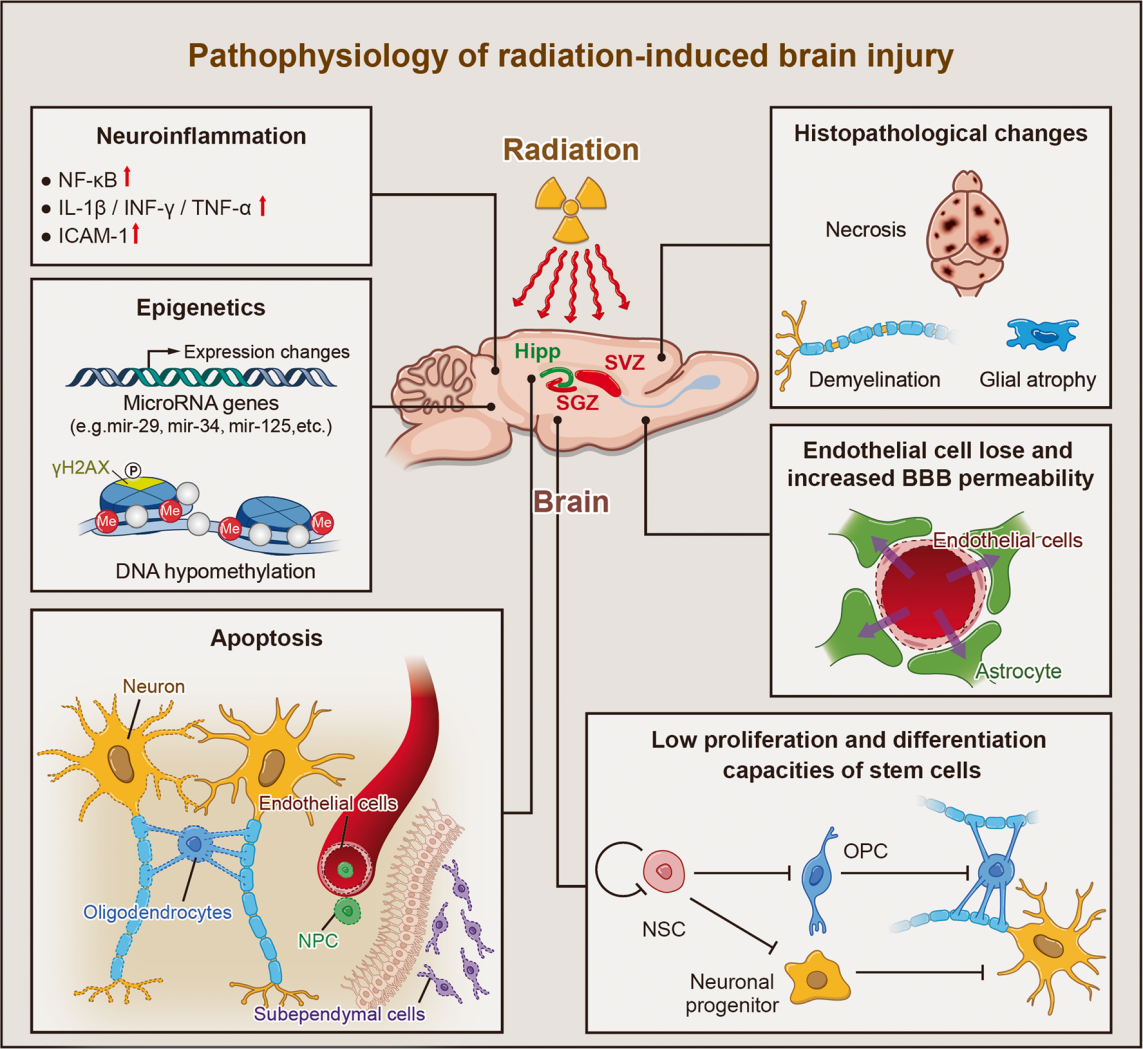
Pathophysiological responses of radiation-induced brain injury. Pathophysiological responses of radiation-induced brain injury include the following. (1) Neuroinflammation associated with increased expression of the transcription factor, NF-κB, as well as upregulated expression of IL-1β, TNF-α, INF-γ, and the adhesion molecule, ICAM-1. (2) Epigenetic alterations associated with changes in the expression levels of microRNAs (e.g., mir-29, mir-34, and mir-125), increased phosphorylation levels of γH2AX, and DNA hypomethylation. (3) Radiation-induced apoptosis of oligodendrocytes, subependymal cells, and certain types of neurons.(4) Low capacities of stem and progenitor cells for proliferation and differentiation. (5) Epithelial cell loss and increased BBB permeability. (6) Histopathological changes, including cell necrosis, glial atrophy, and demyelination. *NSC* neural stem cell, *OPC* oligodendrocyte progenitor cell

Rats are used most frequently to generate animal models of radiation-induced brain injury. Radiation is delivered over the whole brain or to a localized region with varying dosages (Table 1) and using a single or fractionated dosing regimen. Neuroinflammation, epigenetic and histopathological changes, cell apoptosis, impaired cell proliferation and differentiation, and other radiation-triggered events can then be observed at the molecular, cellular, and tissue levels (Fig. 1). Radiation dose, fractionation, and volume play a key role in radiation injury. Besides, radiation dose rate is also a factor which cannot be neglected in evaluating radiation injury caused by a certain dose. The principle dose-rate effect is observed between the low dose-rate and the high dose-rate [6]. Low dose-rate always leads to a reduced damage. Two processes play a key role: intracellular lesion repairment and cell proliferation [7]. Because these processes mainly involve cellular radiobiology, they are well investigated in in vitro cell lines [8]. Dose-rate effect in in vivo cell system was investigated by evaluating the lethality, little radiation dose-rate effects were observed from 0.8 to 1600 Gy/min when LD50/30 or the life-shortening effect in mice were evaluated [9].

**Table 1:** Animal models of radiation-induced brain injury

Animal	Location	Radiation	Dose	Duration	Impact
Rat	Whole brain	Single dose	≤5 Gy	Within 24 h	Apoptosis initiated immediately after irradiation and peaking at 6 h post-exposure, with the numbers of apoptotic cells reaching a plateau at ~3 Gy (the dying cell count did not increase in a radiation dose-dependent manner). A vigorous but transient increase in the numbers of proliferating cells [28], along with impaired neurogenesis [99].
			6–10 Gy	≤4 weeks	Impaired non-matching-to-sample task behavior, but only with relatively long intervals between sample and test trials. Decreased hippocampal neurogenesis and cell proliferation [100]. Increased mRNA and protein expression levels of TNF-α, IL-1, IL-1β, IL-6, and monocyte chemoattractant protein-1 in the hippocampus and cortex, together with increased expression levels of the pro-inflammatory transcription factors, activator protein-1, NF-κB, and cAMP response element-binding protein (CREB) [19]. Significantly increased numbers of apoptotic cells and significantly decreased numbers of proliferating cells [101]. Transient apoptosis of glial cells [26] but no effect on cell viability in another study [10].
				>4 weeks	Long-lasting, decreased proliferation and neurogenesis in the adult brain, along with acute reactive gliosis [37] and decreased hippocampal neurogenesis [102]. Precursor cell proliferation and neurogenesis almost entirely ablated in one study [29], but another study reported no pathologic changes, cognitive impairment, or BBB disruption [83].
			15 Gy	≤4 weeks	Astrocytic GFAP levels in the cortex region slightly elevated, along with increased COX-2 and IL-1β expression levels [10], augmented BBB permeability, enhanced neuronal apoptosis, and increased numbers of activated astrocytes [103]. Cell viability decreased by more than 20 % [10].
			20 Gy	≤4 weeks	Apoptotic rate of glial cells dose-dependently increased at 1 h after irradiation, peaking at 4 h, and then returning to basal levels at 24 h [26]. Transient impairment of cognitive function [83].
				>4 weeks	Decreased hippocampal neurogenesis, cell proliferation, and brain-derived neurotrophic factor (BDNF)/phosphorylated CREB signaling [104]. Cognitive function returned to normal at 60 days post-exposure, with no pathological changes. Normal brain water content despite increased BBB permeability [83]. Vascular lesions (fibrinoid necrosis and hyaline changes) observed. No white matter necrosis [43].
			25 Gy	>4 weeks	Substantial impairment assessed in the water maze test, marked necrosis of the fimbria and degeneration of the corpus callosum, with damage to the callosum [105]. Demyelination with or without necrosis, mainly in the body of corpus callosum and the parietal white matter near the corpus callosum [106].
			30 Gy	≤4 weeks	Reduced numbers of new neurons by 67% and decreased long-term neuronal survival. 51 % reduction in BDNF levels [107].
				>4 weeks	Acute cognitive impairment, reduced numbers (almost absent) of new neurons, and decreased long-term survival of neurons. 33 % reduction in BDNF levels [107].
			40 Gy	≤4 weeks	Mild histopathologic alternations (a somewhat loose and irregular arrangement of neurons together with vascular degeneration in the parietal white matter near the cortex), severe cognitive impairment, increased brain water content, and BBB permeability [83].
				>4 weeks	Severe cognitive impairment, increased brain water content, and BBB permeability [83].
Mouse	Whole brain	Single dose	≤5 Gy	≤4 weeks	Increased numbers of apoptotic cells. Proliferating cells reduced by 75 %, immature neurons in the SGZ reduced by 36 % [60]. Transient upregulation of COX-2, normal neurogenesis [108]. Slightly increased mRNA levels of ICAM-1 [52].
				>4 weeks	Reduced numbers of immature neurons by varying degrees [52]. Hippocampus-dependent memory dysfunction [5].
			6–10 Gy	≤4 weeks	Many pyknotic and dying cells observed in the SGZ and the inner layers of the granule cell layer. Increased average distance between vessels and the nearest doublecortin-positive cell, and reduced numbers of immature neurons and proliferating cells in the SGZ. COX-2, ICAM-1, hypoxia inducible factor-1α, TNF-α, and CCL2 expression levels in the hippocampus significantly increased. VEGF and VEGF receptor 2 levels significantly decreased [52, 109].
				>4 weeks	Depression-like behavior, persistent impaired neurogenesis, and decreased numbers of microglia [108]. Dose-related increases in numbers of activated microglia [60], increased ICAM-1 expression [110], impaired spatial learning and memory, significant hippocampal-dependent cognitive impairments [5], and decreased numbers of adult-born neurons [111]. Depression-like behavior, persistent impaired neurogenesis, and significantly downregulated expression levels of inducible nitric oxide synthase, COX-2, BDNF, and glial-derived neurotrophic factor in the hippocampus [108]. Increased ICAM-1 expression, axonal swelling, and focal demyelination randomly scattered in white matter tracks. BBB disruption. impaired neurogenesis [110].
			11–20 Gy	≤4 weeks	ICAM-1 and TNF-α mRNA and protein levels at a plateau [52]. Significantly enhanced tissue hypoxia [74].
				>4 weeks	Microvessel dilatation in the corpus callosum [110], vascular lesions and fibrinoid necrosis [76], and transient tissue hypoxia [74].
			35 Gy	1 year	No histological changes observed [61].

#### Radiation Dosages

##### Neuroinflammation

Radiation potently stimulates neuroinflammation in the rodent central nervous system (CNS), with significantly increased numbers of activated microglia in the neurogenic zone of the dentate gyrus (DG) of the hippocampus [10]. Radiation induces a pro-inflammatory environment through the activation of inflammation-related genes and over-expression of pro-inflammatory factors in the CNS. Pro-inflammatory factors, including nuclear transcription factor kappa B (NF-κB), assorted cell adhesion molecules, and various cytokines (e.g., interleukin-1 beta (IL-1β), interferon-gamma (INF-γ), and tumor necrosis factor-alpha (TNF-α)) are upregulated within hours after irradiation [10, 11]. These factors affect BBB permeability, leukocyte adhesion, microvessel diameter, and reactive astrogliosis, and feature predominantly in acute radiation-induced brain injury [12–15]. NF-κB regulates cytokine production, including that of IL-1β and INF-γ, which in turn elicits demyelination and edema. TNF-α is associated with hypoxia and contributes to radiation-induced reactive astrogliosis and vascular damage to the BBB. TNF-α also upregulates the expression of intracellular adhesion molecule 1 (ICAM-1). ICAM-1 then allows activated leukocyte entry into the CNS following brain injury.

During the acute injury phase, whole brain radiation at a dose of 10 Gy increases IL-1β and IFN-γ content as early as 4 h after exposure. Expression levels peak at 12 h and then begin to decline toward basal levels at 24 h [16]. Whole brain radiation administered at a single dose of 0.5 Gy with ^56^Fe particles or at 15 Gy with X-rays also acutely increases activated microglia numbers and negatively affect cell survival and neurogenesis [10, 17, 18]. Local brain irradiation at a dose of 20 Gy upregulates ICAM-1 and TNF-α expression levels at 24 h post-exposure, whereas during the chronic injury phase, whole brain radiation at 10 Gy increases TNF-α content for up to 6 months. This may contribute to late radiation-induced injury effects, as suggested previously [19], including cognitive impairment.

##### Epigenetic Alterations

Ionizing radiation causes epigenetic alterations, including DNA methylation, histone modifications, and small RNA-mediated silencing. DNA methylation normally regulates various biological processes and controls tissue-specific gene expression. Radiation is known to induce DNA hypomethylation due to potential radiation-induced DNA damage, altered de novo methyltransferase DNMT3a and DNMT3b expression/activity, as well as radiation-induced persistent induction of reactive oxygen species [20, 21]. DNA hypomethylation is induced in rodents after whole body irradiation with 1 Gy [21]. However, another study conducted by Tawa revealed that whole body x-radiation with 4, 7, and 10 Gy did not change the level of methylated deoxycytidine [22]. Histone modification plays a key role in transcriptional regulation following DNA hypomethylation, and notably, radiation induces phosphorylation of gamma-H2A histone family, member X (γH2AX), along with accumulation of DNA double-stranded breaks. On the other hand, the impact of small RNA-mediated silencing in the radiation response is largely unexplored. A recent study showed that ionizing radiation exerted tissue-, time-, and gender-specific effects on microRNA (miR) expression. Importantly, the miR-29 family was exclusively downregulated in the frontal cortex, resulting in upregulation of methyltransferase DNMT3a [21, 23].

##### Radiation-Induced Apoptosis

Radiation-induced apoptotic cell death reportedly appears in the rodent CNS between 3 and 4 h after exposure and peaks at 6–12 h [24, 25]. Affected cells include oligodendrocytes, subependymal cells, certain types of neurons, endothelial cells, and neural precursor cells in the hippocampal DG. Among these, oligodendrocytes are particularly vulnerable to radiation-stimulated apoptosis. These cells maintain myelin sheaths to insulate neuronal axons, and therefore, radiation-induced oligodendrocyte death is associated with demyelination. Shinohara et al. [25] demonstrated that rat oligodendrocyte numbers decreased within 24 h after a single dose of radiation at 3 Gy, or a total dose of 4.5 Gy with fractionated brain irradiation. The apoptotic rate was time- and dose-dependent and peaked within 8 h of treatment [26]. The apoptotic index of subependymal cells also rapidly increased after whole brain radiation at doses between 0.5 and 2 Gy and peaked at 6 h after exposure to doses of 2–30 Gy [25]. The apoptotic rate was somewhat dose-dependent, with half-maximal cell death observed within seven dose fractionations at 1.5 Gy per fraction. No further apoptosis was observed after four fractions [25]. Elsewhere, microglial cells were activated with a single radiation dose of 8 Gy at 6 h after brain irradiation, but the cell population decreased 7 days later [27].

##### Impaired Stem Cell Proliferation and Differentiation

Stem and progenitor cells possess the capacity to proliferate and differentiate and thus are extremely vulnerable to ionizing radiation. Mature neurons within most brain regions lose their proliferative capacity and are considered to be in a permanent state of growth arrest. However, stem and progenitor cell proliferation and differentiation occur throughout life in the subgranular zone (SGZ) of the hippocampal DG, the subventricular zone (SVZ) of the lateral ventricular wall, and the olfactory bulb but are reduced by cranial irradiation [28].

Monje et al. showed that precursor cell growth was impaired after whole brain radiation at 2 Gy in a dose-dependent manner [29]. In another study, oligodendritic progenitor cell numbers were reduced by fractionated radiation at 50 Gy administered over ten fractions [30]. Other reports suggest that decreased neurogenesis following ionizing radiation may contribute to impaired cognitive function [31–36]. Radiation instigates a long-lasting decrease in immature and proliferative cell numbers after cranial exposure to X-rays at doses as low as 6 Gy within 9 weeks after radiation [37]. The potential mechanisms behind the reduction in proliferative cell numbers include acute ablation of the progenitor cell population, impaired growth potential, and alterations in the neurogenic microenvironment [29]. In addition to altered proliferation capacity, the ability of neural stem cells and oligodendrocyte progenitors to differentiate into neurons and glial cells was also impaired by ionizing radiation after treatment with whole brain radiation at 2 Gy [29].

##### Endothelial Cells and BBB Disruption

Radiation to the brain has a profound effect on the vasculature. Radiation-induced brain injury is partially due to vascular system damage, resulting in late onset brain necrosis. Radiation reduces endothelial cell numbers, vessel density, and vessel length when administered in 5Gy/fraction (eight fractions in total) at 10–52 weeks after exposure [38]. Moreover, radiation dose-dependently induces endothelial apoptosis. For example, a single dose of 5 Gy with local brain irradiation caused a 15 % decrease in endothelial cell numbers at 24 h after exposure, cell loss was maintained for at least 1 month [39]. These findings are consistent with a study showing that BBB permeability and vascular endothelial growth factor (VEGF) expression increased within 24 h after exposure to radiation at 6 Gy and that the increases were maintained for 1 month [40]. Doses higher than 5 Gy (e.g., local radiation of 25 Gy) led to pronounced endothelial cell loss after 24 h [41].

##### Histopathological Changes

Radiation dose is one of the most important factors determining the severity of damage and the latent period between irradiation and the occurrence of lesions. Radiation-induced brain injuries include glial atrophy, demyelination, necrosis, varying degrees of vascular changes, and other histopathological alterations. Radiation doses higher than 20 Gy will certainly cause cerebrovascular lesions [42, 43]. In several studies, Wistar albino rats were irradiated with single dose, whole brain radiation, with doses ranging from 10 to 40 Gy. Rats exposed to radiation at 10 Gy showed no significant histopathological or clinical changes until death, nor did they exhibit a shortened survival period. However, cerebrovascular lesions without necrosis were observed at 15 months post-exposure in rats irradiated with 20 Gy. The predominant vascular lesions involved hyaline thickening and fibrinoid necrosis of vessel walls, as well as microaneurysms. In rats receiving doses of 30 and 40 Gy, the major changes included large necrotic lesions occurring mainly in the white matter, especially the internal capsule and the fimbria of the hippocampus. Hence, changes in brain white matter are time- and dose-dependent [3, 42, 44].

The most radiosensitive components of the brain are proliferating cells and immature neurons in the SVZ and SGZ, while the fimbria is the most sensitive brain region in the white matter to X-rays, followed by the corpus callosum and the internal capsule. Calvo et al. [3] locally irradiated the rat brain with a single dose of X-rays ranging from 17.5 to 25 Gy and only observed white matter necrosis and demyelination at intervals of ≥39 weeks and after doses of ≥22.5 Gy.

#### Radiation Dose Fractionation

Radiation-induced brain injuries are also related to dose fractionation. Both large single doses and fractionated doses are of great use in clinical practice. Large single doses are delivered to patients during stereotactic radiosurgery, while fractionated schedules are used during standard radiotherapy protocols. Hornsey et al. [44] reported an iso-effect curve for X-ray-induced brain damage in rats by comparing the effects of dose fractionation into 8, 15, or 30 equal fractions given in the same overall treatment time of 6 weeks. The curve showed that target cells were capable of repairing a larger amount of radiation damage as the number of fractions increased, indicating that increased dose fractionation is an effective way of sparing damage to the CNS.

Lamproglou et al. [45] established a model of radiation-induced cognitive dysfunction by radiating the whole brain of elderly rats at a dose of 30 Gy divided into ten fractions given over 12 days. Seven months after exposure, the rats showed signs of cognitive dysfunction, but no pathological abnormalities in the brain were observed at the light microscopic level. Similarly, radiation divided into eight fractions of 5 Gy per fraction led to chronic and progressive cognitive dysfunction from 26 weeks to at least 1 year after exposure, but no gross histopathological changes were observed [46, 47]. Other studies showed that doses higher than 25 Gy, which are expected to cause radiation necrosis when applied in a single fraction, yielded no necrosis when distributed over more than one fraction [38, 48–50]. Indeed, incidences of radiation-induced necrosis of only 5 and 10 % are predicted at biologically effective doses of 120 and 150 Gy, respectively, for fractionated irradiation of the rodent brain with a fraction size of <2.5 Gy [51]. Gaber et al. [52] concluded that molecular responses (e.g., modulation of ICAM-1 and TNF-α expression) to single-dose radiation are rapid, whereas corresponding responses to fractionated radiation are slow.

### Radiation Volume

Radiation volume of the brain also dictates the consequences of radiation exposure. The severity of radiation-induced injury increases as the volume of the irradiated tissue increases. Münter et al. [42] irradiated rats with 2 and 3 mm collimators and single radiation doses ranging from 20 to 100 Gy and found that animals irradiated with the 3 mm collimator had a higher incidence of late structural changes in the brain than those irradiated with the 2 mm collimator. These data are consistent with those of Carger et al. [53], who showed that larger radiation volume is related to higher risk and more severe radiation-induced brain injury. However, different elements within the same organ can show varying radio-responsiveness [54], and the relationship between volume and severity of radiation injury is complicated and depends on the anatomical and physiological properties of the irradiated region. Previous studies have also suggested that partial brain radiation may engender less severe cognitive impairment than whole brain radiation or large field radiation [55, 56].

### Mice

The mouse is the most similar rodent species to the human and, like the rat, is also widely used to develop radiation-induced brain injury models. Mice are advantageous in that they (1) are relatively easy to maintain and breed, (2) are easily handled, and (3) show ~70 % homology with humans at the genetic level. However, mice are disadvantageous in that (1) the small size of the brain makes the organ difficult to accurately locate, resulting in uneven dose distribution and damage to the respiratory system and (2) mice are too fragile to undergo repeated anesthesia when long-term observation periods are required.

#### Radiation Doses

##### Neuroinflammation

Radiation of the brain results in multiple inflammatory changes, as exemplified by increased expression of cytokines and glial cell activation. Both acute and chronic neuroinflammatory responses are detected in irradiated brains and may serve as therapeutic targets for the successful use of corticosteroids and cyclooxygenase-2 (COX-2) inhibitors to treat radiation-induced injury [57, 58]. During the acute injury phase, whole body radiation with 7 Gy stimulated TNF-α and IL-1β mRNA and protein expression in the brain, with peaks at 2–8 h after exposure [59]. Expression levels of TNF-α and IL-1β returned to normal by 24 h. In another study, whole brain radiation with 10 Gy dose-dependently increased the numbers of activated microglia at 2 months after exposure [60]. Doses of 15 Gy or higher resulted in increased mRNA levels of CC chemokine ligand 2 (CCL2), IL1-α, and TNF-α at 4 h and 1 day after radiation [61], while radiation with 20 Gy (but not 8 Gy) resulted in astrocyte activation as early as 4 h and as late as 1 year after exposure [62]. Whole brain radiation of 35 Gy elevated COX-2 and prostaglandin E2 expression at 4 h. These changes were related to radiation-induced brain edema.

During the chronic injury phase, radiation at ≥15 Gy resulted in the dose-dependent activation of microglia 1 month later. Elevated CCL2, glial fibrillary acidic protein (GFAP), hemeoxygenase-1 (HO-1), and TNF-α levels were detected with radiation at 25 Gy 6 months after exposure. Increased ICAM-1 levels and numbers of CD11c-positive/major histocompatibility complex II-positive cells, indicative of a mature dendritic cell phenotype, were maintained for 1 year after irradiation [61].

##### Radiation-Induced Apoptosis

As noted above, radiation-induced apoptotic cells include oligodendrocytes, subependymal cells, some neurons, and neural precursor cells of the hippocampal DG. A single dose of radiation at 2 Gy induced apoptosis of neural precursors, but not of neurons, within 24 h post-exposure in the developing mouse brain [63]. Similarly, whole brain radiation at 2 Gy induced clusters of apoptotic cells in the subependymal region, which peaked at 4 h and decreased to baseline levels at 24 h post-radiation [64]. The time course of radiation-induced apoptosis in the subependymal region of mice was similar to that in the rat CNS [65]. Whole brain radiation at 5 Gy induced apoptosis in the white but not gray matter of the brain at 8 h post-radiation, while 25 Gy applied to the midbrain resulted in little or no apoptosis at 2 h [66]. The discrepancy may be due to varying radiosensitivity in different brain regions [67].

##### Neurogenesis and Cognitive Impairment

The “neurogenesis zone” is the most radiosensitive part of the brain, and radiation-induced changes occur here at low doses without evident histological abnormalities. The neurogenesis zone includes the SGZ of the hippocampal DG and the SVZ along the lateral ventricles. Radiation at 2 Gy affects neurogenesis but not gliogenesis at 3 weeks post-exposure [60]. Furthermore, single-dose radiation at 2–10 Gy yields dose-dependent, persistent apoptosis in the SGZ [5, 60, 68–70]. Because cells within the SGZ are responsible for cognitive function [5, 29, 68, 71], this might explain why animal models can develop cognitive dysfunction with no obvious histological changes.

##### Endothelial Cell Loss and BBB Disruption

Previous studies have investigated the relationship between radiation dose, endothelial cell loss due to apoptosis, and BBB permeability. In one study, brain irradiation led to hippocampal endothelial cell loss at radiation doses as low as 0.5 Gy [72]. In another study, a total dose of 40 Gy at 2 Gy per fraction was delivered to mice to evaluate radiation-induced BBB permeability. BBB permeability increased at 90 days after fractionated radiation and was maintained for up until 180 days, while astrocyte proliferation began to increase at 60 days post-exposure [73]. However, single dose, whole brain radiation at 20 Gy augmented BBB permeability at 24 and 48 h post-exposure, and also decreased arteriole diameter at 48 h after radiation treatment [10]. Moreover, single dose, unilateral brain irradiation at 20 Gy altered the structure and function of microvascular networks in both the irradiated and the unexposed hemisphere [15, 74].

##### Mortality Rates and Histopathological Changes

As noted above, radiation dose crucially affects the risk and severity of radiation injury. Chiang et al. [75] irradiated the left brain hemisphere of C3H strain mice within a 1.5 × 0.5 cm region using single-dose radiation at doses ranging from 2 to 60 Gy. Dose-response survival curves were plotted at 300 days post-exposure. Consequently, the median lethal dose (LD50) was calculated as 32.4 Gy. Contrarily, a single dose of 20 Gy dose-dependently decreased myelin-associated protein content, but induced no necrotic lesions or mortality within 4–6 months. Therefore, the doses used to generate mouse models of radiation injury are recommended to be <32.4 Gy. On the other hand, whole brain radiation of C57BL/6 mice at 35 Gy failed to show increased mortality within 1 year of exposure due to brain injury [61]. The different mortality rates with approximately the same dose of radiation may stem from strain-specific brain radiosensitivity or other factors.

In terms of histopathological changes, vascular lesions and necrosis were observed with whole brain radiation of mice at 13 Gy between 41 and 87 weeks after exposure [76]. Additionally, radiation at a total dose of 60 Gy at 6 Gy per fraction delivered to the left hemisphere of BALB/c mice elicited localized necrosis at 3 months post-exposure [77].

#### Fractionated Radiation

Fractionated irradiation is commonly employed to generate radiation-induced brain injury animal models. Yuan et al. [73] irradiated mice with a daily dose of 2 Gy for a total dose of 40 Gy. The mouse model displayed increased BBB permeability and ultrastructural changes in the brain and was therefore useful for elucidating mechanisms of radiation-induced injury. Amulya et al. [78] employed brain radiation at 4 Gy per fraction for a total dose of 20 Gy, resulting in persistent cognitive dysfunction in young mice related to reduced numbers of proliferating and immature neurons in the hippocampus. This model is similar to the clinical situation especially that of pediatric cancer patients receiving fractionated brain irradiation. In another study, radiation at 4.5 Gy per fraction for a total dose of 36 Gy caused impaired contextual learning at 1 month post-exposure, deficits in spatial learning at 2 months post-exposure, and decreased vascular density at 4 months post-exposure. The behavioral changes were maintained over an observation period of 5 months [79]. Furthermore, whole brain radiation with a single dose of 20 Gy led to pronounced increases in ICAM-1 and TNF-α mRNA and protein expression levels, while a total dose of 40 Gy at 2 Gy per fraction caused only mild increases [52]. Therefore, as noted in the rat section of this review, cellular and molecular responses to single-dose irradiation are rapid, whereas responses to fractionated irradiation are relatively slow.

#### Radiation Volume

As described above, the risk and severity of radiation injury increase as the volume of the irradiated area increases. Radiosurgery is usually well tolerated when the radiation volume is small [80]. For example, whole brain radiation at 5 Gy induced apoptotic cell death in the white matter of the brain at 8 h after exposure, while localized midbrain irradiation at 25 Gy resulted in little or no apoptosis [66, 67]. On the other hand, unilateral brain irradiation resulted in the elimination of proliferating cells from the ipsilateral hemisphere. Proliferating cell numbers decreased to ~10–20 % in the granule cell layer of the contralateral hemispheres at 12 h after irradiation with 4–12 Gy [81]. This is similar to another study in which whole brain radiation at 2–10 Gy induced a 2–75 % reduction in proliferating cell numbers at 48 h post-exposure [5]. However, the proliferating cells reappeared after recovery periods of 24 h and 7 days, especially in the SVZ.

## Discussion and Conclusions

Establishment of an appropriate animal model is useful for the study of the mechanisms and prevention of radiation-induced brain injury and optimization of therapeutic strategies for the treatment of damage stemming from radiotherapy. Here, we reviewed various rat and mouse models employed in previous research and discussed the pros and cons of the rodent models, as well as radiation doses, volume, and fractionation.

Rats and mice are commonly used to produce animal models of radiation-induced brain injury, in addition to many other disease and injury states. These animals are relatively easy to breed and maintain and show genetic similarities with humans. However, the small size of the mouse brain limits the radiation volume and dose distribution and yields relatively low survival rates after anesthesia and irradiation. Therefore, rats are utilized more frequently than mice in radiation-based studies.

Radiation dose is also of utmost importance when considering the risk of radiation-induced brain injury. Radiation doses must be considered when generating radiation-induced brain injury models. The tolerance of the brain also limits the practical dose. For example, the LD50 is 32.4 Gy for mouse [75] and 33 Gy for rat [3]. Radiation dose applied in animal model was expected to below the LD50. Radiation at 20 Gy causes vascular lesions at 15 months post-exposure, whereas radiation at 22.5 Gy induces the formation of necrotic lesions at only 39 weeks post-exposure in the rat [3]. Doses higher than 22.5 Gy would clearly lead to necrosis with a shorter period of latency. For large single exposures, radiation doses at 10, 20, and 25 Gy are associated with reduced mortality relative to larger doses but still provoke pathognomonic symptoms. Therefore, these doses are widely used by researchers to investigate radiation-mediated damage [26, 59, 82–88]. Doses higher than 2 Gy cause cell apoptosis and inflammation in the SVZ and SGZ, without obvious histopathological changes in their radiated region [19]. Doses of <10 Gy are used to study changes in proliferating and immature cells [28].

High dose, single fraction radiation is typically used to generate animal models of acute white matter necrosis [77, 89–92], while fractionated radiation and lower radiation doses are used to generate models of cognitive dysfunction [93–98]. With regard to fractionated brain radiation, exposures of 2 and 4 Gy per fraction are frequently used in animal studies, because these doses mimic those employed in clinical protocols. Irradiated tissues are more capable of repairing damage when receiving fractionated radiation, such that subjects undergoing treatment can tolerate a relatively higher total radiation dose without suffering radiation necrosis.

Ample evidence supports a positive relationship between radiation-induced damage and irradiated volume, where large brain field radiation is associated with a higher risk of serious radiation-induced brain injury.

The incidence and severity of radiation-induced brain injury depend not only on radiation dose, fractionation, volume, and radiation dose-rate, but also on attendant chemotherapy, age, concomitant diabetes, and spatial factors. Further studies are required to precisely determine the parameters of the dose-response relationship in terms of radiation volume and fractionation. The results of these studies will greatly contribute to optimized treatment strategies for patients undergoing brain and neck radiation.
